# Role of the Nuclear Receptor Corepressor 1 (NCOR1) in Atherosclerosis and Associated Immunometabolic Diseases

**DOI:** 10.3389/fimmu.2020.569358

**Published:** 2020-10-08

**Authors:** Martin A. Geiger, Ana T. Guillaumon, Francesco Paneni, Christian M. Matter, Sokrates Stein

**Affiliations:** ^1^ Vascular Diseases Discipline, Clinics Hospital of the University of Campinas, Campinas, Brazil; ^2^ Center for Molecular Cardiology, University of Zurich, Schlieren, Switzerland; ^3^ Department of Cardiology, University Heart Center, University Hospital Zurich, Zurich, Switzerland; ^4^ Department of Research and Education, University Hospital Zurich, Zurich, Switzerland

**Keywords:** ****atherosclerosis, ****cardiometabolic, corepressor complex, NCoR1, nuclear receptor signaling, immunometabolism, mechanisms of disease, transcriptional regulation

## Abstract

Atherosclerotic cardiovascular disease is part of chronic immunometabolic disorders such as type 2 diabetes and nonalcoholic fatty liver disease. Their common risk factors comprise hypertension, insulin resistance, visceral obesity, and dyslipidemias, such as hypercholesterolemia and hypertriglyceridemia, which are part of the metabolic syndrome. Immunometabolic diseases include chronic pathologies that are affected by both metabolic and inflammatory triggers and mediators. Important and challenging questions in this context are to reveal how metabolic triggers and their downstream signaling affect inflammatory processes and vice-versa. Along these lines, specific nuclear receptors sense changes in lipid metabolism and in turn induce downstream inflammatory and metabolic processes. The transcriptional activity of these nuclear receptors is regulated by the nuclear receptor corepressors (NCORs), including NCOR1. In this review we describe the function of NCOR1 as a central immunometabolic regulator and focus on its role in atherosclerosis and associated immunometabolic diseases.

## Introduction

**Figure 1:**
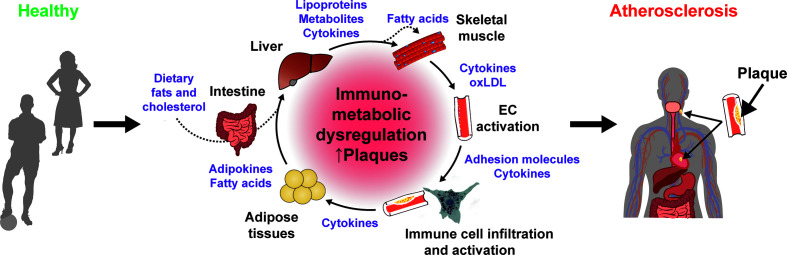
Immunometabolic dysregulation promotes atherogenesis. Contribution of different organs and immunometabolic mediators to the chronic dysregulation that promote atherogenesis.

Atherosclerosis is characterized by the accumulation of immune cells, cholesterol species and other lipids in the intimal space of arteries. The disease primarily affects large, elastic, and high-pressure vessels, such as the coronary, renal, femoral, and carotid arteries. The complex pathophysiology is triggered by genetic and environmental risk factors, including lipid species and metabolites (e.g., cholesterol, specific fatty acids, carnitine), as well as by hypertension, diabetes and obesity (1–3). Molecular, genetic, dietary and pharmacological studies over the last decades suggest that hyperlipidemia, especially hypercholesterolemia, combined with the genetic predisposition is a major trigger of atherogenesis (4–9). Nevertheless, recent research showed that other metabolic and inflammatory processes are closely interconnected at the cellular level as well as *via* intra- and inter-organ communication (10–12) (**Figure 1**).

## Immunometabolic Integrators

The signaling cascades that are activated by inflammatory and metabolic triggers and/or mediators converge at key transcriptional regulators, which in turn coordinate the expression of specific target genes and atherogenic processes. Whether individual target genes are activated or repressed depends on several other factors, such as folding and compaction of the chromatin, posttranslational modifications of histones by chromatin-modifying enzymes, functional alterations by noncoding RNAs, and recruitment of the transcriptional machinery, including transcription factors and importantly also transcription cofactors, i.e. transcriptional corepressors and coactivators (13–16).

### Lipid-Responsive Nuclear Receptors With Immunometabolic Functions

Nuclear receptors are a large family of druggable transcription factors that exert important functions in development, metabolism, and immune response (17, 18). Consequently, several nuclear receptors do affect the development of atherosclerosis and associated cardiometabolic diseases (reviewed in (19)). While the role of lipid-binding nuclear receptor in metabolism is well established, studies over the last decade demonstrated that several of these nuclear receptors, including PPARγ, LXRs, and LRH-1, mediate transrepression of pro-inflammatory molecules in the liver and/or immune cells, such as macrophages and T cells (20–25). Therefore, these nuclear receptors act as direct immunometabolic regulators and play an important role in atherogenesis.

### NCOR1—An Emerging Regulator of Immunometabolic Processes

Given their central role to integrate upstream information and regulate the expression of downstream target genes, specific transcriptional cofactors function as central immunometabolic regulators. About 300 transcription cofactors are known to exist in mice and human cells (26). However, only a fraction of those is expressed in a specific cell type or tissue, and their function is restricted to certain pathways and transcription factors (14, 15). Some of these factors are involved in inflammatory mechanisms, others known to exert metabolic functions.

**Figure 2:**
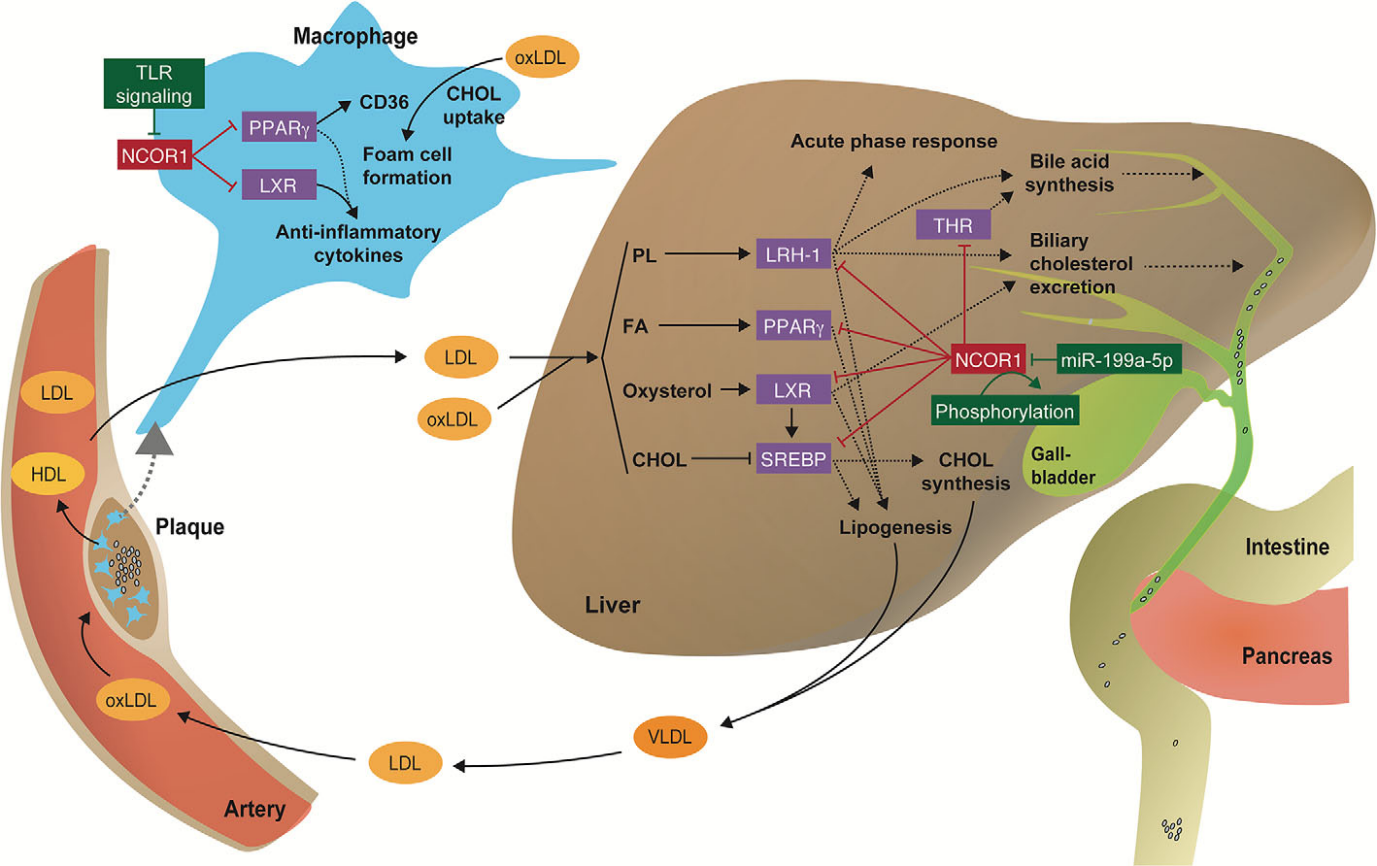
Regulation of immunometabolic processes by the NCOR1 complex. Scheme illustrating how transcriptional corepressor complexes repress the function of nuclear receptors and thus regulate key inflammatory and metabolic processes in the liver and macrophages that are involved in the pathophysiology of cardiometabolic diseases. CHOL, cholesterol; FA, fatty acid; oxLDL, oxidized LDL; PL, phospholipid.

Could research on (anti-)atherogenic corepressor complexes provide answers to the key question of causality between metabolic and inflammatory changes? One large nuclear receptor corepressor complex that has been studied extensively *in vitro* and *in vivo* in the last years is nuclear receptor corepressor 1 (NCOR1) (**Figure 2**).

NCOR1 serves as scaffolding protein that forms the basis for a large corepressor complex, including protein deacetylases [including class I (HDAC3), class II (HDAC4, 5, 7, and 9) and class III (SIRT1) KDACs], transducin beta-like 1 (TBL1) and TBLR1, two highly related F box/WD40-containing factors, and the G-protein-pathway suppressor 2 (GPS2) (27, 28). The molecular functions and (patho-)physiological role of HDAC3, class II HDACs, GPS2, and TBL1 has been extensively reviewed (29–31). Although germline *Ncor1* ^-/-^ and *Ncor2* ^-/-^ mice are embryonically lethal (32, 33), they enabled to establish important roles for NCOR1 in erythropoiesis, T-cell, and central nervous system development, whereas NCOR2 contributes to the morphological development of the heart (32–34). Targeted deletions of NCOR1 in immune cells, liver, adipose tissue, and muscle demonstrated that it affects pro- and anti-inflammatory gene signatures, mitochondrial function, lipid metabolism, insulin sensitivity, intestinal cholesterol absorption, thereby highlighting its immunometabolic functions, which will be discussed below (21, 35–41). To the best of our knowledge there are no studies addressing the tissue-specific deletion of *Ncor2*, which is essential to study the cell-specific function of a gene, especially for genes that are expressed in nearly all cells and organs and thus regulate multiple processes (e.g., a detailed expression pattern of *Ncor1* and *Ncor2* in mice can be found at *Tabula muris* (https://tabula-muris.ds.czbiohub.org). Therefore, this review will focus on tissue-specific functions of NCOR1.

### NCOR1 Complex in Macrophages

Monocyte-derived macrophages are the most abundant immune cell population found in atherosclerotic lesions and plaques. They take up oxidized LDL (oxLDL) in an uncontrolled fashion, secrete pro-inflammatory cytokines that recruit T cells, and fall apart in a necrotic fashion, thus promoting plaque growth and destabilization (42, 43). Silencing of macrophage NCOR1 *in vitro* produces the same phenotype of activated macrophages: increased expression pro-inflammatory cytokines, chemokines and metalloproteases, and enhanced macrophage invasiveness (21). Mechanistically, it was proposed that the NCOR1 corepressor complex acts as a transcriptional checkpoint for these inflammatory genes: In the absence of a nuclear receptor ligand, NCOR1 is recruited to target genes and interacts with unphosphorylated c-Jun bound to target promoters, thus repressing pro-inflammatory gene expression. Upon stimulation with an innate inflammatory stimulus, such as LPS, a structural change in the ligand-binding domain of the nuclear receptor reduces its affinity to NCOR1, hence simultaneously increasing the affinity to coactivators (44). TBLR1 plays a crucial function as an E3-ligase that directs the ligand-dependent ubiquitination and proteasome-mediated clearance of NCOR1 and histone deacetylase 3 (HDAC3) from the promoter of target genes (45). Moreover, pro-atherosclerotic minimally oxidized LDL (oxLDL) promotes JNK-dependent derepression of AP-1 by releasing NCOR1 from the promoter of chemokine genes (40). Taken together, these *in vitro* studies suggest that absence of NCOR1 exacerbates the inflammatory response in macrophages.

Paradoxically, the *in vivo* myeloid cell-specific deletion of *Ncor1* repressed inflammatory gene expression and improved insulin sensitivity in a diet-induced obesity mouse model (39). Li et al. suggest that deletion of *Ncor1* in adipose tissue macrophages leads to a derepression of LXR target genes, increased expression of *de novo* lipogenesis and fatty acid desaturation genes, and subsequent production of anti-inflammatory ω3 fatty acids, which in turn suppress inflammatory activation of macrophages (39) (**Figure 2**).

Using a similar loss-of-function approach, Oppi et al. recently showed that the myeloid cell-specific deletion of *Ncor1* increased atherosclerosis at the aortic sinus and the thoraco-abdominal aorta in *Ldlr* knockout mice (41). At the molecular level NCOR1 was found to bind to the *Cd36* promoter thus repressing PPARγ-driven *Cd36* expression. Consequently, peritoneal macrophages from *Ncor1*-deficient mice had increased CD36-mediated oxLDL accumulation and foam cell formation (**Figure 2**). Interestingly, *Ncor1*-deficient macrophages displayed an increased expression of both anti- and pro-inflammatory genes. Moreover, analyses of omics-datasets obtained from human plaque specimens suggested that NCOR1-driven PPARγ suppression is also protective in human plaque development and vulnerability (41).

The data from Oppi et al. and Li et al. are partially controversial (39, 41). Two major reasons could explain the differences. First, the genetic background of the mice: Myeloid cell-specific Ncor1 knockout mice on a ‘wildtype’ versus *Ldlr*-/- background. The *Ldlr* deficiency shifts the lipoprotein metabolism towards a humanized profile with very high LDL-cholesterol levels, which makes this mouse model prone to develop atherosclerosis and display a pro-inflammatory signature (9, 46). Second, the use of the experimental diets: High-fat diet to study the impact of NCOR1 on diet-induced obesity versus high-cholesterol diet to assess atherogenesis. It is known from numerous studies that diets differently affect major immune and metabolic processes and hence the development of chronic immunometabolic diseases, especially when investigating lipid-sensitive nuclear receptor signaling such as LRH-1 (47–51).

The phenotype of myeloid cell-specific *Hdac3* knockout mice is partially resembling the data from Oppi et al. (41, 52). Hoeksema et al. showed that *Hdac3*-deficient mice develop more atherosclerotic plaques in the aortic sinus compared to control mice (52). Furthermore, *Hdac3*-deficient macrophages displayed an anti-inflammatory wound-healing like phenotype. On the other side, atherosclerotic plaques from myeloid cell-specific *Hdac3* knockout mice displayed increased collagen disposition and enlarged protective fibrous caps, while the myeloid cell-specific *Ncor1* knockout plaques had increased necrotic cores (41).

In line with the data from Oppi et al. (41), Fan et al. showed that myeloid cell-specific *Gps2*-knockout mice display an increased expression of pro-inflammatory cytokines and chemokines, such as *Ccl2* and *Ccl7*, which is characteristic of pro-inflammatory M1-type macrophage activation (53). Moreover, the authors used palmitate as obesity-linked metabolic trigger of inflammation and observed that *Gps2*-deficient macrophages had an elevated pro-inflammatory gene signature (53). Vice-versa, transplantation of *Gps2*-overexpressing bone marrow into two mouse models of obesity reduced inflammation and improved insulin sensitivity in recipient mice (53). Moreover, docking of GPS2 and LXR stimulate H3K9 demethylation on the *Abcg1* promoter, thus promoting its expression and mediating cholesterol efflux to HDL particles in monocytic THP-1 cells (54). Contrary to the induction of *Abcg1 via* LXR, GPS2 interacts with NF-κB to promote the expression of *Abca1* upon LPS stimulation (55). These elegant studies demonstrate how GPS2 promotes macrophage cholesterol efflux upon different stimuli, i.e. *via* oxysterol-triggered LXR activation or *via* LPS-driven NF-κB activation. Interestingly, the activation of both processes by GPS2 is independent of NCOR1-HDAC3 recruitment (54, 55).

Aside from its effects on atherosclerotic plaques and metabolism, macrophage NCOR1 seems to play a pivotal role in the heart. Genetic deletion of *Ncor1* in macrophages led to reduced infarct size and improved cardiac function in mice with experimental myocardial infarction (56). These findings were explained by suppression of inflammatory transcriptional programs (interleukin‐1β, interleukin‐6, AP-1 and nuclear factor‐κB) and reduced macrophage proliferation (due to inhibition of cell cycle progression). Hence, macrophage NCOR1 may act as an upstream regulator of myocardial inflammation thus participating to left ventricular hypertrophy, diastolic dysfunction and microvascular disease, key hallmarks of heart failure (57). In the same study, macrophage NCOR1 deficiency markedly inhibited neointimal hyperplasia and vascular remodeling in a mouse model of arterial wire injury (56). Taken together, these results suggest that selective modulation of NCOR1 in macrophages could have important implications for the prevention of myocardial ischemic damage, heart failure, coronary heart disease and intracoronary stent restenosis. Further molecular work and preclinical studies are needed to better explore the potential of NCOR targeting approaches in cardiovascular disease.

### NCOR1 Complex in the Liver

The liver plays a crucial role in the development of atherosclerosis by regulating metabolic and inflammatory processes, such as the expression of pro-inflammatory cytokines and acute phase response proteins, the secretion of VLDL particles, the uptake of cholesterol from the circulation, and the biliary cholesterol excretion. An immunometabolic dysregulation in the liver can promote nonalcoholic fatty liver disease and the development of atherosclerosis. Importantly, nonalcoholic fatty liver disease leads to adverse cardiovascular functions, such as increased oxidative stress and endothelial dysfunction, hypercoagulability, and accelerated development of atherosclerosis (58–60).

Several studies addressed the physiological functions of NCOR1, NCOR2, and HDAC3 in the liver, which target several lipid-responsive nuclear receptors (**Figure 2**) (24, 28, 29, 61–67). The role of NCOR1 in liver energy metabolism is particularly interesting during the fasting-feeding transition: both HDAC3 and NCOR1 are known to repress lipogenic genes, but paradoxically, NCOR1 was also reported to be critical for inhibition of PPARα, hepatic fatty oxidation and ketogenesis (63, 64). This is due to the ability of NCOR1 to select its repressor targets in a context-dependent manner to orchestrate liver energy metabolism depending on the energy status of the cell (37). Upon feeding, high levels of glucose and insulin activate the target of rapamycin complex 1 (mTORC1)-AKT signaling pathway, thus phosphorylating serine 1460 of NCOR1 (pS1460 NCOR1) (**Figure 2**). pS1460 decreases the ability of NCOR1 to interact with LXR, thus promoting the transcription of lipogenic LXRs target genes, and conversely, by fostering the interaction with PPARα and ERRα, with subsequent repression of downstream ketogenic and Oxphos genes (37).

Besides being regulated by phosphorylation in the liver, the translation of NCOR1 can also be blocked by the microRNA miR-199a-5p (68) (**Figure 2**). The authors used a bioinformatic approach to identify miRNAs affected in a non-alcoholic steatohepatitis (NASH) model induced by a methionine-choline−deficient (MCD) diet. The MCD diet increased miR-199a-5p expression, which in turn blocks the translation of the *Ncor1* mRNA by binding to a conserved 3’ untranslated region (68).

To assess the function of NCOR1 in thyroid hormone receptor (TR) regulation, the group of A. Hollenberg generated a truncated form of NCOR1 which lacks the two main nuclear receptor interacting domains (NCOR1ΔID) and thus, for example, cannot interact with TRs or LXRs (69, 70). Interestingly, they found that mice with a disrupted nuclear receptor binding domain show a reduced content of cholesterol in the liver and an increased synthesis of alternative bile acid. In turn, these less hydrophobic bile acids have a lower capacity to bind fats and sterols in the intestine and thus reduce their absorption (38). The increased expression of alternative bile acid synthesis genes is a consequence of TRβ1 de-repression caused by the mutant NCOR1 (38).

GPS2 exerts protective function in the liver by interacting with SUMOylated nuclear receptors, such as LRH-1 and LXRβ, to repress inflammatory cytokine expression during the hepatic acute phase response (71). On the other side, hepatic deletion of GPS2 reduces nonalcoholic steatohepatitis *via* induction of PPARα-driven lipid catabolism (67). This repression of PPARα happens in cooperation with NCOR1, but not NCOR2 (67). Conversely to the GPS2 knockouts, hepatic deletion of TBL1 promotes hypertriglyceridemia and hepatic steatosis on both a normal chow or high-fat diet by impairing PPARα-driven lipid catabolism (72). The induction of PPARα was mediated by increased recruitment of NCOR1-HDAC3 complexes in the absence of TBL1 (72). These data underline that the repressive function and target specificity of the NCOR1 complex is largely dependent on its co-regulators.

### NCOR1 in Cardiomyocytes

Previous studies demonstrated that NCOR1 deficiency increases the activity of MEF2d in skeletal muscle (35). In a recent publication, Li et al. affirmed the suppressive role of NCOR1 in regulating the size of cardiomyocytes, presenting strong evidence of interactions among NCOR1, MEF2, and class IIa HDACs, being MEF2a and MEF2d key transcription factors interceded the impact of NCOR1 on cardiomyocyte size (73). As Li et al. described, NCOR1 may be considered as a stress-responsive and cardioprotective regulator during cardiac hypertrophy, showing that its deficiency led to cardiac hypertrophy under physiological condition and aggravated hypertrophy induced by pressure overload (73).

NCOR1 usually cooperates with HDACs to execute its repressive activities (28). The regulatory paradigm in cardiac hypertrophy involves alterations in gene expression that is mediated by chromatin remodeling. HDACs remove the acetyl group from histones, resulting in its hypoacetylation, which diminishes chromatin accessibility for transcription factors, leading to repression of transcription (74). The Class I HDAC3 enzyme participates in the repressive activities of NCOR1, and its deficiency in cardiomyocytes causes severe cardiac hypertrophy at an early stage. Interestingly, Li et al. identified class IIa to be involved in the process of cardiomyocytes, demonstrating that NCOR1 works more likely through class IIa instead of class I to affect cardiac hypertrophy (73).

### BCL6/NCOR1-Mediated Repression of Inflammation

B cell lymphoma-6 (BCL6) belongs to a class of zinc-finger transcription factors and acts as a transcriptional repressor. It regulates the development of germinal centers, B and T cells, coordinates the activation of macrophages, and was described as a proto-oncogene (75). Importantly, the interaction of BCL6 with NCOR complexes is essential to mediate its transrepressive activity in several biological processes (75, 76).

The PPARδ agonist GW0742 protects against angiotensin II (AngII)-accelerated atherosclerosis by inducing the expression of the *Bcl6*, and the regulators of G protein-coupled signaling (RGS) proteins RGS4 and RGS5, which in turn inhibit the expression of pro-inflammatory and atherogenic genes (77). Interestingly, ChIP-seq data demonstrate that the BCL6 and NCORs cistrome overlap in about 50% of all DNA binding sites, and that binding sites that are synergistically bound by BCL6 and NCORs are highly enriched for inflammatory and atherogenic NF-κB target genes (78).

The deletion of *Bcl6* in the liver leads to a derepression of PPARα-driven enzymes mediating fatty acid oxidation and thus protects against high-fat diet-induced hepatic steatosis (79). Interestingly, binding of the corepressors NCOR1, NCOR2 and HDAC3 to BCL6-binding sites was reduced in *Bcl6*-deficient livers, and these sites displayed increased enhancer/promoter activity as shown by enhanced histone 3 lysine 27 acetylation (H3K27ac) (79). These data suggest that hepatic BCL6 recruits a subset of NCOR/HADC3 complexes to the promoter of specific target genes regulating lipid metabolism.

### Potential Functions of the NCOR1 Complex in Endothelial and T Cells

So far, the role of NCOR1 using an endothelial cell-specific genetic model has not been explored. Therefore, this section is limited to describe the function of its tight cofactor HDAC3 (28). HDAC3 is essential for endothelial monolayer survival and integrity. Studies demonstrated that HDAC3 is engaged in the differentiation of embryonic stem cells into endothelial progenitors and determinant for endothelial cell survival (64). Zampetaki et al. demonstrated that disturbed flow induces transient stabilization of the HDAC3 protein in endothelial cells. It happens through the activation of the VEGFR2 and PI3 kinase signaling pathways (80). HDAC3 expression increases near branch openings compared with areas of high laminar flow, which was also confirmed by exposure of endothelial cells to disturbed flow *in vitro* (80). It is therefore likely that NCOR1 might be involved in these processes as well.

To obtain a good overview about the function of NCOR1 in T cells we refer to an excellent review from Muller et al. (81). Most studies assessed the role of NCOR1 during T cell development, while only a few focused on its function in mature T cells in pathophysiological conditions. The lethal phenotype of the complete *Ncor1* knockout mice developed by Jepsen et al. demonstrated that constitutive *Ncor1*-deficiency conferred impaired thymocyte development (33). Further studies using T cell-specific deletions of *Ncor1* in mice revealed that the number of double negative thymocytes and peripheral T cells are decreased in the absence of NCOR1 (82, 83). Recently, it was shown that NCOR1 regulates the function of CD4+ and Th1 cells by regulating the expression of IFN-γ (84). Consistently, Zhang et al. showed that deletion of *Ncor1* in male SJL T cells disrupted the fenofibrate-driven repression of IFN-γ synthesis, possibly also explaining differences in Th1 responses between male and female mice (85). Another interesting report demonstrated that NCOR1 represses the tolerogenic program in dendritic cells. As a consequence, dendritic cell-specific *Ncor1* knockout mice display an increased number of FoxP3+ regulatory T cells (86). In terms of atherogenesis, this could turn into a protective effect.

## Perspectives

Research over the past decades identified various molecular factors that affect atherogenesis, including inflammatory and metabolic regulators (2, 87, 88). In the last years, we have learned that immunometabolic pathways are often interconnected, and we are now starting to understand the major factors involved in these immunometabolic mechanisms (10, 11). Targeting immunometabolic pathways, e.g., those regulated by NCOR1, might become an attractive approach to prevent the development of atherosclerosis since they regulate various processes that promote the development of the disease. Moreover, since these pathways are also implicated in the development of other chronic immunometabolic diseases, such as obesity, type 2 diabetes, and nonalcoholic fatty liver disease, their mechanistic dissection will be relevant to understand development and progression of immunometabolic diseases on a broader scale.

Which could be suitable strategies to target NCOR1? One option would be to develop small molecule agonists or antagonists that interfere or facilitate the interaction of NCOR1 to a specific target nuclear receptor. Although this would be a specific approach, it would be very laborious and time-consuming. Another option is offered by current developments in gene therapy (including CRISPR therapeutics) and small interference RNA (siRNA) medicine, which offer excellent future targeting opportunities. The liver is an easily accessible target organ and new emerging drugs using siRNAs demonstrate that transcribed RNA can be targeted in a liver-specific fashion by conjugating the synthetic siRNA to triantennary N-acetylgalactosamine carbohydrates (e.g., as it is used to target *PCSK9* in the liver (89, 90)). However, specifically targeting other organs or cells, such as cardiomyocytes and macrophages, continues to be challenging. Moreover, the identification of atherogenic targets, such as CD36 in macrophages (41), might lead to the identification of druggable targets and hence to the development of new therapeutic strategies to treat atherosclerotic disease patients that are at a very high risk to suffer a transient ischemic attack, stroke or acute coronary syndrome.

## Author Contributions

All authors analyzed the literature and wrote the manuscript. All authors contributed to the article and approved the submitted version.

## Funding

SS was supported by the Swiss National Science Foundation (PZOOP3_161521), the SwissLife Jubiläumsstiftung, the Swiss Heart Foundation (FF19025), and the Foundation for Research in Science and the Humanities at the University of Zurich (STWF-20-008).

## Conflict of Interest

The authors declare that the research was conducted in the absence of any commercial or financial relationships that could be construed as a potential conflict of interest.
